# Sugar activation and glycosylation in Plasmodium

**DOI:** 10.1186/s12936-015-0949-z

**Published:** 2015-10-31

**Authors:** Marta Cova, João A. Rodrigues, Terry K. Smith, Luis Izquierdo

**Affiliations:** ISGlobal, Barcelona Ctr. Int. Health Res. (CRESIB), Hospital Clínic, Universitat de Barcelona, Barcelona, Spain; Instituto de Medicina Molecular, Faculdade de Medicina da Universidade de Lisboa, Av. Prof. Egas Moniz, Edificio Egas Moniz, 1649-028 Lisbon, Portugal; Biomedical Sciences Research Complex, University of St Andrews, North Haugh, St Andrews, Fife, KY16 9ST UK

**Keywords:** Glycobiology, Malaria, Plasmodium falciparum, Sugar nucleotides

## Abstract

Glycoconjugates are important mediators of host-pathogen interactions and are usually very abundant in the surface of many protozoan parasites. However, in the particular case of *Plasmodium* species, previous works show that glycosylphosphatidylinositol anchor modifications, and to an unknown extent, a severely truncated N-glycosylation are the only glycosylation processes taking place in the parasite. Nevertheless, a detailed analysis of the parasite genome and the recent identification of the sugar nucleotide precursors biosynthesized by *Plasmodium falciparum* support a picture in which several overlooked, albeit not very prominent glycosylations may be occurring during the parasite life cycle. In this work, the authors review recent developments in the characterization of the biosynthesis of glycosylation precursors in the parasite, focusing on the outline of the possible fates of these precursors.

## Background

The cell surfaces and endosomal/lysosomal systems of protozoan parasites are usually rich in glycoconjugates, some of which play essential roles in their survival, infectivity or virulence [1]. In *Plasmodium falciparum*, the only glycan structures described so far are limited to glycosylphosphatidylinositol (GPI) anchors [2–5] and, recently, to unusual N-glycans composed of one or two GlcNAc residues [6, 7]. The glycan structures of *P. falciparum* GPI anchors are well characterized [3]. However, controversial questions regarding the glycobiology of *P. falciparum*, such as the presence of O-glycosylation or the extent and significance of N-glycosylation, remain open [8, 9].

In the blood stages, *P. falciparum* primarily relies upon glycolysis for its energetic requirements [10]. Due to the need of a large amount of glucose, *P. falciparum* increases the hexose permeability of the red blood cell (RBC) membrane by expressing an essential hexose transporter at the surface of the infected RBC [11, 12]. The presence of active sugar nucleotide biosynthetic routes in the parasite indicates that there is a flux of glucose for the synthesis of these various glycosylation precursors [13–16]. Sugar nucleotides can be synthetized, in general, through two main pathways: a *de novo* pathway, which involves inter-conversion of an existing sugar or sugar nucleotide, and a salvage pathway, which relies upon “activation” of the sugar by a kinase and a subsequent pyrophosphorylase to form a sugar nucleotide [15]. Thus, despite that evolution into a parasitic niche seems to have resulted in “paring down” of many *Plasmodium* metabolic pathways, the presence of sugar nucleotides suggests an involvement in the biosynthesis of different parasite glycans [10]. The biosynthesis of GDP-fucose and other sugar nucleotides not related to GPI anchors strongly suggests a role in the biosynthesis of glycans/glycolipids that are not yet characterized in the parasite [6, 8, 17–19].

## GDP-mannose

**Fig. 1:**
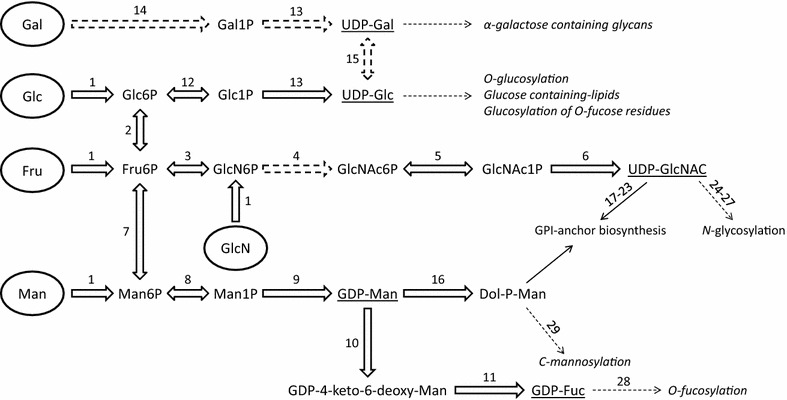
Sugar nucleotide biosynthesis pathways in *Plasmodium falciparum*. Activated sugars, used for glycoconjugate biosynthesis, are underlined. Carbohydrates, taken up from the medium or salvaged, are circled. Sugar nucleotide donor fates are indicated and they are in *italics* when glycosylations have not been proved to be present in the parasite. The numbers refer to the enzymatic activities summarized in Table 1. Discontinuous arrows depict enzymatic activities that could not be identified in *P. falciparum* genome

GDP-mannose (GDP-Man), the activated form of mannose, is biosynthesized in a multistep process from mannose salvaging or via a *de novo* pathway from fructose-6-phosphate (Fru6P). Metabolic databases, based on the parasite genome sequence, predict the conservation of both biosynthetic routes (Fig. 1) [13]. In the *de novo* pathway a mannose-1-phosphate isomerase (MPI; EC 5.3.1.8) catalyzes the interconversion of Fru6P to mannose-6-phosphate (Man6P). Two more enzymes catalyze the conversion of Man6P into GDP-Man, firstly phosphomannomutase (PMM; EC 5.4.2.8) forms mannose-1-phosphate (Man1P), which is then converted into GDP-Man by a Man1P guanyltransferase (MPG; EC 2.7.7.13). The salvage pathway comprises the phosphorylation of mannose into Man6P by a hexokinase (HK; EC 2.7.1.1), after which the pathway follows the same route as the *de novo* pathway (see Table 1) [13, 15]. The presence of a mannose salvage pathway has been demonstrated through the incorporation of [^3^H]Man into GPI anchors by the blood stages of the parasite [2, 15, 20].

**Table 1:** Enzymes involved in sugar nucleotide pathways, GPI-anchor and C-, N- and O-glycan biosynthesis of *P. falciparum*

Step	Enzyme name	Enzyme number	*P. falciparum* homologues^a^	Syntenic orthologs^b^
1	Hexokinase (HK)	EC 2.7.1.1	PF3D7_0624000	Pv, Pk, Pc, Pr, Pb, Py, Pch
2	Glucose-6-phosphate isomerase (G6PI)	EC 5.3.1.9	PF3D7_1436000	Pv, Pk, Pc, Pr, Pb, Py, Pch
3	Glucosamine-fructose-6-phosphate aminotransferase (GFPT)	EC 2.6.1.16	PF3D7_1025100	Pv, Pk, Pc, Pr, Pb, Py, Pch
4	Glucosamine-phosphate N- acetyltransferase (GNA)	EC 2.3.1.4	No gene identified	
5	Phosphoacetylglucosamine mutase (PAGM)	EC 5.4.2.3	PF3D7_1130000	Pv, Pk, Pc, Pr, Pb, Py, Pch
6	UDP-N-acetylglucosamine pyrophosphorylase (UAP)	EC 2.7.7.23	PF3D7_1343600	Pv, Pk, Pc, Pr, Pb, Py, Pch
7	Mannose-6-phosphate isomerase (MPI)	EC 5.3.1.8	PF3D7_0801800	Pv, Pk, Pc, Pr, Pb, Py, Pch
8	Phosphomannomutase (PMM)	EC 5.4.2.8	PF3D7_1017400	Pv, Pk, Pc, Pr, Pb, Py, Pch
9	Mannose-1-phosphate guanyltransferase (MPG)	EC 2.7.7.13	PF3D7_1420900	Pv, Pk, Pc, Pr, Pb, Py, Pch
10	GDP-mannose 4,6-dehydratase (GMD)	EC 4.2.1.47	*PF3D7_0813800*	Pv, Pk, Pc, Pr, Pb, Py, Pch
11	GDP-L-fucose synthase (FS)	EC 1.1.1.271	*PF3D7_1014000*	Pv, Pk, Pc, Pr, Pb, Py, Pch
12	Phosphoglucomutase (PGM)	EC 5.4.2.2	PF3D7_1012500	Pv, Pk, Pc, Pr, Pb, Py, Pch
13	UTP-glucose-1-phosphate uridylyltransferase (UGP) or UDP-sugar pyrophosphorylase (USP)	EC 2.7.7.9 or EC 2.7.7.64	PF3D7_0517500	Pv, Pk, Pc, Pr, Pb, Py, Pch
14	Galactokinase (GK)	EC 2.7.1.6	No gene identified	
15	UDP-glucose 4-epimerase (GALE)	EC 5.1.3.2	No gene identified	
16	Dolichol-phosphate mannosyltransferase polypeptide 1 (DPM1)	EC 2.4.1.83	PF3D7_1141600	Pv, Pk, Pc, Pr, Pb, Py, Pch
Enzymes involved in GPI-Anchor biosynthesis				
17	phosphatidylinositol n- acetylglucosaminyltransferase (PIG-A)	EC 2.4.1.198	PF3D7_0618900.1 and PF3D7_0935300 and/or PF3D7_1032400 and/or PF3D7_1141400	Pv, Pk, Pc, Pr, Pb, Py, Pch
18	N-acetylglucosaminyl phosphatidylinositol deacetylase (PIG-L)	EC 3.5.1.89	PF3D7_0624700 and/or PF3D7_0911000	Pv, Pk, Pc, Pr, Pb, Py, Pch
19	Inositol acyltransferase (PIG-W)	EC 2.3	PF3D7_0615300	Pv, Pk, Pc, Pr, Pb, Py, Pch
20	GPI mannosyltransferase I (PIG-M)	EC 2.4.1	PF3D7_1210900	Pv, Pk, Pc, Pr, Pb, Py, Pch
21	GPI mannosyltransferase II (PIG-V)	EC 2.4.1	PF3D7_1247300	Pv, Pk, Pc, Pr, Pb, Py, Pch
22	GPI mannosyltransferase III (PIG-B)	EC 2.4.1	PF3D7_1341600	Pv, Pk, Pc, Pr, Pb, Py, Pch
23	GPI mannosyltransferase IV	EC 2.4.1	No gene identified	
Enzymes involved in N-glycans biosynthesis				
24	UDP-N-Acetyl-glucosamine-1-P transferase (ALG7)	EC 2.7.8.15	PF3D7_0321200	Pv, Pk, Pc, Pr, Pb, Py, Pch
25	UDP-N-Acetylglucosaminyltransferase subunit (ALG13)	EC 2.4.1.141	PF3D7_0806400	Pv, Pk, Pc, Pr, Pb, Py, Pch
26	UDP-N-Acetylglucosaminyltransferase subunit (ALG14)	EC 2.4.1.141	PF3D7_0211600	Pv, Pk, Pr, Pb, Py, Pch
27	Catalytic subunit of the oligosaccharyltransferase complex (STT3)	EC 2.4.99.18	PF3D7_1116600	Pv, Pk, Pc, Pr, Pb, Py, Pch
Enzymes involved in O-fucosylation				
28	GDP-fucose protein O-fucosyltransferase 2 (PoFUT2)	EC 2.4.1.221	PF3D7_0909200	Pv, Pk, Pc, Pr, Pb, Py, Pch
Enzymes involved in C-mannosylation				
29	C-mannosyltransferase	EC 2.4.1	PF3D7_0806200	Pv, Pk, Pc, Pr, Pb, Py, Pch

^a^All the gene ID numbers are identified and annotated in *P.* *falciparum* genome as putative candidates. The genes in italics (GMD and FS) are the only ones that have been functionally characterized [12]

^b^Syntenic orthologs identified in other *Plasmodium* species. *Pv* (*P. vivax*), *Pk* (*P. knowlesii*), *Pc* (*P. cynomolgy*), *Pr* (*P. reichenowi*), *Pb* (*P. berghei*), *Py* (*P. yoelii*) and *Pch* (*P. chabaudi*) [22]

**Fig. 2:**
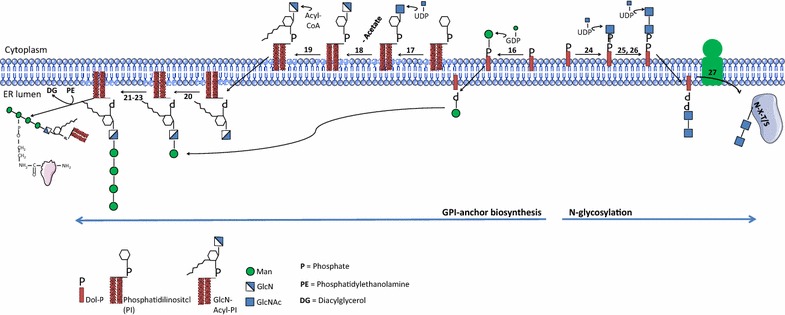
Biosynthetic scheme for GPI-anchor biosynthesis and N-glycosylation in *Plasmodium falciparum*. The numbers refer to the enzymes described in Table 1

In eukaryotes, Man is an important constituent of N-, O-linked glycans and glycosylphosphatidylinositol (GPI) anchors. However, Man residues are absent in *P. falciparum* N-glycans, as the parasite synthesizes a severely truncated N-glycan precursor composed of one or two GlcNAc residues (Fig. 2) [2, 6, 7]. Nonetheless, Man residues are present in the *P. falciparum* major glycoconjugates, the GPI anchors that play an important role in the pathogenicity of the parasite. GPIs are attached to the C-terminus of many important surface proteins, such as MSP-1, and anchor them to the external leaflet of the plasma membrane. Besides, in the surface of the parasite there are also protein-free GPIs that function as malarial toxins and are involved in parasite-induced release of cytokines such as tumor necrosis factor (TNF) and interleukin 1 (IL-1) [3, 9, 21–24].

**Fig. 3:**
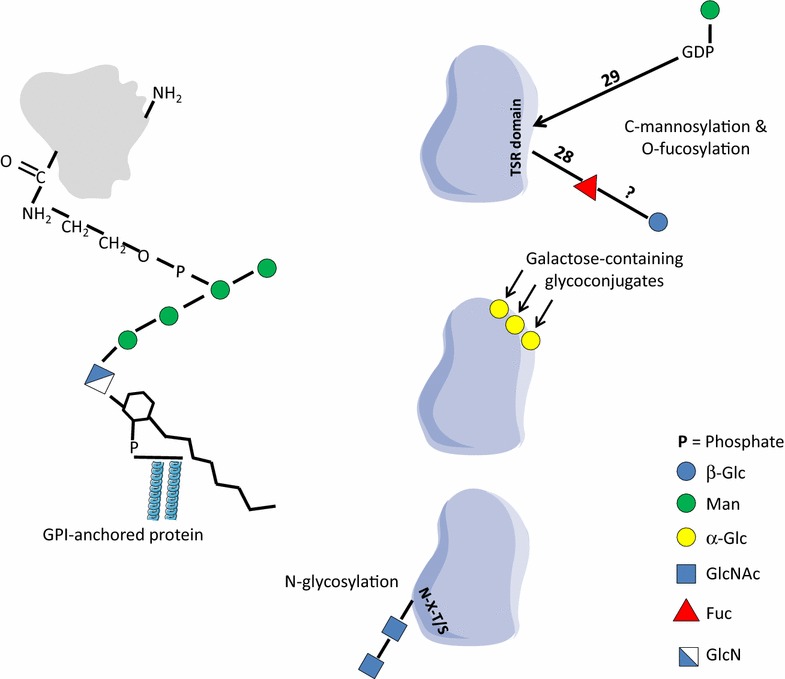
Schematic representation of described or putative glycosylations present in the surface of the malaria parasite. Enzymatic activities predicted to be involved in the glycosylation reactions described are indicated by numbers and summarized in Table 1

*Plasmodium falciparum* GPIs consist of the conserved glycan core (Manα1-2Manα1-6Manα1-4GlcNH_2_α1-6*myo*-Ins) but have an extra/forth mannose and inositol-acylation. Thus, the complete *P. falciparum* GPI structure is defined as EtNP-6(Manα1-2)Manα1-2Manα1-6Manα1-4GlcNα1-6(acyl-2)*myo*-Ins-1-P-(*sn*1,2 diacyl)-glycerol (Figs. 2, 3) [2, 3, 20, 25]. The protein free GPI glycan core contains either 3 or 4 mannose residues since two structurally distinct GPI-anchor precursors (*Pf*α and *Pf*β) are used by the parasite. *Pf*α presents an additional α1,2-mannose residue modifying the terminal mannose of the conserved trimannosyl core glycan [2, 3, 20, 21]. The biosynthesis of *P. falciparum* GPI anchors as with all other eukaryotes, starts with the preassembly of a GPI precursor in the cytoplasmic face of the ER membrane. Briefly, the addition of GlcNAc to phosphatidylinositol (PI) by phosphatidylinositol glycosyltransferase-A (PIG-A) gives rise to GlcNAc-PI (Fig. 2), which is then de-N-acetylated to form GlcN-PI by a de-N-acetylase (PIG-L)(steps are discussed in detail below). Prior to mannosylation at the 4 position of the GlcN, an inositol acyltransferase (PIG-W) transfers a fatty acid (usually myristate or palmitate) from acyl-CoA to the 2-OH group of the D-*myo*-inositol residue of GlcN-PI (Fig. 2). Subsequently, the GPI precursor is translocated from the cytoplasmic to the luminal face of the ER, where four Man residues are added sequentially by four different GPI-mannosyltransferases (Fig. 2). The Man donor for these mannosyltransferases is dolichol-phosphate-mannose (Dol-P-Man) formed by the action of dolichol-phosphate mannose polypeptide 1 (known as DPM1) alternatively known as Dol-P-Man synthase (DPMS). Interestingly, *P. falciparum* DPMS represents a unique class in the clade of DPMS enzymes [26] that has been genetically validated as essential (Williams and Smith, manuscript in preparation). Dol-P-Man is formed through the coupling of Man from GDP-Man to Dol-P to form Dol-P-Man and GDP as by-product. Three of the four mannosyltransferases required for GPI-biosynthesis, PIG-M, PIG-V and PIG-B encoding putative Man1, Man2 and Man3 transferases respectively, can be identified in the parasite genome [13, 27, 28]. However, no clear candidate genes for addition of Man4 (also performed in yeast by the *Smp3* gene) are found [27]. Interestingly, a recent study suggests that *P. falciparum* PIG-B is responsible of adding the extra Man to the GPI precursor [29].

Several essential proteins present in the surface of various stages of the malaria life cycle, such as *Pfs*48/45 on gametes, *Pfs*25 on ookinetes, circumsporozoite (CS) on sporozoites and MSP-1 or MSP-2 on merozoites, are GPI-anchored proteins [30]. Therefore, GPI anchors play important roles in *P. falciparum* survival and pathogenicity. The essentiality of GPI-anchored proteins is supported by genetic studies. For instance, *Pfs*48/45 gene disruption prevents zygote development and transmission whereas mutant parasites lacking CS protein do not form sporozoites [31, 32]. In the blood stages, it was demonstrated that six proteins out of seven merozoite GPI-anchored proteins were refractory to genetic deletion, strongly suggesting an essential role in parasite survival [33].

Another possible fate for GDP-Man/Dol-P-Man is the C-mannosylation of proteins [34]. C-mannosylation is the attachment of a Man residue to tryptophan through a carbon–carbon bond. This type of glycosylation is found in WXXW sequences of secreted proteins and cell surface receptors containing thrombospondin type I repeat (TSR) domains [35]. Whereas TSR has been recognized as critical for protein adhesion and recognition in several organisms including *Plasmodium* [36, 37], the direct influence of C-mannosylation on protein function is not known. The enzyme responsible for C-mannosylation is a C-mannosyltransferase, which was first identified as DPY-19 in *Caenorhabditis elegans* [38]. In *P. falciparum* genome there is a DPY-19 homolog, which is present in all sequenced *Plasmodium spp*. genomes. Further investigations are required to confirm the presence/activity of this putative C-mannosyltransferase and the biological significance of this post-translational modification in *P. falciparum*.

## GDP-fucose

Fucosylation is important in a wide variety of organisms, as it is associated with numerous types of recognition and adhesion events. GDP-fucose (GDP-Fuc), the precursor for all the fucosylation reactions, has been identified in the blood stages of *P. falciparum* at levels similar to the pools present in other protozoan parasites [15, 39]. The *P. falciparum* genome contains homologues of the enzymes involved in the *de novo* biosynthesis of GDP-Fuc from GDP-Man (Fig. 1 and Table 1) [13]. This pathway relies in a three-step conversion of GDP-Man to GDP-Fuc catalyzed by two enzymes: a GDP-Man dehydratase (GMD; EC 4.2.1.47) and a GDP-Fuc synthetase (FS; EC 1.1.1.271) also known as GDP-4-dehydro-6-deoxy-d-mannose epimerase/reductase. *Pf*GMD and *Pf*FS are expressed throughout the intraerythrocytic life cycle and have been shown to be active in in vitro studies [15]. In some organisms a salvage pathway for the biosynthesis of GDP-Fuc is also present. This pathway involves the phosphorylation of fucose by a fucose kinase (EC 2.7.1.52) followed by condensation with GTP catalyzed by a fucose-1-phosphate pyrophosphorylase (EC 2.7.7.30) [40]. However, *P. falciparum* genome lacks obvious candidate genes for these enzymatic activities and [^3^H]fucose is not significantly incorporated by the parasite, supporting the idea that most of the GDP-Fuc in the blood stages of *P. falciparum* is formed through the *de novo* biosynthetic pathway, i.e. conversion from GDP-Man (Fig. 1 and Table 1) [2, 13, 15]. Nevertheless, the utilization of GDP-[^3^H]Fuc by *P. falciparum* lysates suggests that the GDP-Fuc donor is used by the parasite in fucosylation reaction(s) as yet unidentified [15, 41].

Fucose has not yet been described in any glycoconjugates from *P. falciparum*. Hence, the fate and importance of GDP-Fuc for *P. falciparum* remains unknown [6, 9]. Our own preliminary data supports the presence of a fucose-containing glycan in the surface of the parasite, since a *Pf*GMD null mutant shows a decreased labelling with fucose-binding *Ulex europaeus* agglutinin I (UEA-I) (Izquierdo and Samuelson, in preparation). Thus, despite the non-essentiality of GDP-Fuc for the growth/replication of the parasite in the blood-stage, it seems that *P. falciparum* presents, at least, a fucosyltransferase activity. The best candidate for a GDP-Fuc dependent glycosyltransferase activity in the parasite is a protein *O*-fucosyltransferase 2 (PoFUT2) homolog conserved in the genome (Table 1). In other organisms, PoFUT2 is involved in the O-fucosylation of TSR domains [42]. Remarkably, there are several TSR domain-containing proteins identified in *P. falciparum* with essential roles for infectivity and survival [37]. For instance, thrombospondin-related anonymous protein (TRAP) is crucial for sporozoite gliding motility and hepatocyte invasion, whereas merozoite TRAP (MTRAP) plays a role as putative adaptor between the merozoite invasion machinery and the surface proteins that mediate erythrocyte adhesion [43, 44]. CS protein, the main component of the RTS,S malaria vaccine, is involved in sporozoite infection and also contains an altered TSR domain [45]. The expression of TRAP and CS protein fragments in HEK293T cells showed that their TSR domains were modified with fucose residues, presumably by PoFUT2 present in HEK293T cells [17, 18]. Interestingly, peptides of *Pf*GMD, *Pf*FS and PoFUT2, the three principal components for GDP-Fuc metabolic route and O-fucosylation machinery, have been detected in the sporozoite stages of the parasite, when the surface of the cell is covered with CS and TRAP [46]. Altogether, the data strongly suggests the presence of an active PoFUT2 mediated O-fucosylation mechanism in sporozoites, which needs further exploration.

## UDP-N-acetyl glucosamine

UDP-N-acetyl glucosamine (UDP-GlcNAc), the donor for all GlcNAc transferases, plays an important role in several eukaryotes. It is essential in *Leishmania major* and in *Trypanosoma brucei* for growth and survival in the mammalian host [47, 48]. There are two main active pathways for UDP-GlcNAc biosynthesis in *P. falciparum*: a conventional *de novo* pathway and a salvage pathway fed by glucosamine (GlcN) (Fig. 1). The *de novo* pathway (the amino-sugar pathway) starts with the conversion of Fructose-6P (Fru6P) into Glucosamine-6P (GlcN6P) through the glucosamine-fructose-6-phosphate aminotransferase activity (GFPT; EC 2.6.1.16) [13, 15]. The next step, which is the acetylation of GlcN6P to generate N-acetyl-glucosamine-6P (GlcNAc6P), remains a mystery in *P. falciparum* since a candidate gene encoding for the glucosamine-phosphate-N-acetyltransferase (GNA) activity (EC 2.3.1.4) cannot be identified in the genome (Table 1). GNA enzymes have been characterized in several eukaryotes, including *S. cerevisiae* and *T. brucei*, but despite their well-conserved secondary structure, the amino acid sequences are often diverse [49, 50]. Besides, the presence of various histone-acetylases makes it challenging to unequivocally identify a *P. falciparum* GNA in the genome. After acetylation, GlcNAc6P is converted into N-acetyl-glucosamine-1P (GlcNAc1P) by a phosphoacetylglucosamine mutase (PAGM, EC 5.4.2.3). The last step is catalyzed by an UDP-N-acetylglucosamine pyrophosphorylase (UAP, EC 2.7.7.23) which converts GlcNAc1P into UDP-GlcNAc [13, 15]. The salvage pathway for UDP-GlcNAc production exists possibly due to the action of hexokinase (HK; EC 2.7.1.1) which catalyzes the phosphorylation of glucosamine (GlcN) to GlcN-6-P which then feeds the same route as the *de novo* pathway (Fig. 1, Table 1) [15]. Several studies demonstrate the existence of this salvage pathway since GPI-anchors can be labelled with [^3^H]GlcN [2, 20, 51]. However, the contribution of this pathway in vivo seems to be minor, as GlcN is not an abundant sugar within the parasite hosts. As in the case of GDP-Man, the UDP-GlcNAc pathway is predicted to be essential in *P. falciparum* as it feeds GPI-anchor biosynthesis, required for survival and infectivity [9].

UDP-GlcNAc is used in *P. falciparum* N-glycosylation. Despite that the presence of N-glycans in parasite proteins was initially controversial [2, 52] recent work show evidences of the presence of short N-glycans on the surface of *P. falciparum* trophozoites and schizonts [6]. This agrees with the synthesis of Dolichol-PP (Dol-PP) linked GlcNAc and GlcNAc_2_ glycan precursors [7] and the conservation in the parasite’s genome of the genes involved in the biosynthesis of *P. falciparum* N-glycans: ALG7 (EC 2.7.8.15), ALG13/ALG14 (EC 2.4.1.141) and STT3 (EC 2.4.99.18) (Table 1). ALG7 transfers GlcNAc, from UDP-GlcNAc, to the ER-membrane Dol-PP forming Dol-PP-GlcNAc [7, 53]. ALG13/ALG14, a heterodimeric UDP-GlcNAc transferase complex, uses UDP-GlcNAc as donor substrate for the extension of Dol-PP-GlcNAc to Dol-PP-GlcNAc_2_. ALG14 acts as a scaffold, recruiting ALG13 (which retains a consensus UDP-sugar binding site such as ALG7) to the cytosolic face of ER where occurs the catalysis of Dol-PP-GlcNAc_2_ [54–56]. STT3, normally part of a 5 subunit complex, comprising of OST1, WBP1, STT3, OST4 and OST3, but OST4 seems to be missing from the complex [52], which catalyzes the transfer of GlcNAc and GlcNAc_2_ from Dol-PP-linked oligosaccharides to “sequon” Asparigine residues (N-X-T/S) in the nascent protein (Fig. 2) [7, 57, 58]. Interestingly, the N-linked glycosylation blocker tunicamycin is lethal for the parasite when it is exposed to the compound for two developmental cycles (more than 48 h), although the authors did not relate that effect to N-linked oligosaccharide biosynthesis [59, 60]. Furthermore, if these short N-glycans elicit a specific immune response in the human host, they may be interesting as xenoantigens since these glycans are not expected to be present in the human glycome.

GlcN, the deacetylated form of GlcNAc, is an integral component of GPI-anchors (Fig. 2). To generate GlcN-PI, two reactions take place in the cytoplasmic side of the ER membrane: the transfer of GlcNAc from the UDP-GlcNAc donor to the phosphatidylinositol (PI) and the deacetylation of GlcNAc-PI to GlcN-PI (Fig. 2) [3, 13, 25, 27]. The first reaction is catalyzed by a phosphatidylinositol N-acetylglucosaminyltransferase (EC 2.4.1.198). In mammals this reaction is associated to a complex of six proteins [27, 61–64] and four subunits are conserved in the genome of the parasite [13]. N-acetylglucosaminyl phosphatidylinositol deacetylase (EC 3.5.1.89) is responsible for the deacetylation of GlcNAc-PI [13, 27]. Two candidate genes (Table 1) show certain homology to PIG-L that encodes for a de-N-acetylase in other organisms. However, the specific gene encoding for PIG-L in *P. falciparum* has not been functionally characterized [13, 65]. Once formed in the cytoplasmic side of the ER, the GlcN-PI GPI-precursor migrates to the luminal side for the addition of the mannose residues. GPI-anchored proteins are crucial for the parasite infectivity, virulence and survival. GPIs are also significant pro-inflammatory endotoxins of *P. falciparum* that, over their release after RBC rupture, induce cytokine and adhesin expression in macrophages and the vascular endothelium that correlates with severe malaria [22, 66, 67].

## UDP-galactose

The incorporation of galactose into glycoproteins and glycolipids in eukaryotic cells is through the activated sugar precursor UDP-galactose (UDP-Gal). UDP-Gal was recently identified in the blood stages of the *P. falciparum* life cycle [15]. A candidate gene for a UDP-glucose 4-epimerase (EC 5.1.3.2) activity that produces UDP-Gal via the epimerization of UDP-glucose has yet to be identified (Table 1) [13]. Therefore, the production of this sugar nucleotide can be performed via activation of galactose 1 phosphate (Gal1P) by Gal1P uridylyltransferase (EC 2.7.7.12) which has also not been identified in the parasite’s genome. Other possibilities are enzymes with a UTP-glucose-1-phosphate uridylyltransferase activity (UGP, EC 2.7.7.9) presenting also a weak galactose-1-phosphate uridylyltransferase activity (EC 2.7.7.10), as occurs in mammals; or a broad substrate range UDP-sugar pyrophosphorylase (USP, EC 2.7.7.64) as described in plants and *Leishmania major* [68–71]. However, although galactose competes for PfHT1 hexose permease [72, 73], there is not a clear galactokinase (EC 2.7.1.6) candidate in the parasite genome. Furthermore, the biological relevance of the UDP-Gal pool is unknown (Fig. 1), and the presence of UDP-Gal and galactose-containing glycoconjugates (either glycolipids and/or glycoproteins) in the parasite remains a controversial issue.

The first evidences of the presence of galactosylated glycoconjugates in *P. falciparum* were reported by Ramasamy and Reese when they showed the reduction of the antigenicity of certain parasite proteins from infected red blood cells after a galactosidase treatment [74, 75]. Furthermore, it was observed that titers of anti-α-gal Abs were significantly elevated in sera collected from subjects living in malaria endemic areas or patients with acute *P. falciparum* malaria in Asia [76]. Maréchal et al. described the incorporation of radiolabelled UDP-Gal by late blood-stage *P. falciparum* lysates [77]. They also explored the presence of galactose-containing glycolipids in the apicoplast membranes, a common trait in plastids from plants and algae [78]. However, a recent lipidomic analysis of the parasite’s organelle confirmed the absence of galactoglycerolipids in *P. falciparum* apicoplast [79]. Ramasamy and Field also demonstrated that terminal α-galactosylation was minimal in *P. falciparum* late asexual blood stages, judging by α-galactose-specific lectin binding and UDP-[^3^H]Gal incorporation [80]. Nevertheless, recently Yilmaz et al. provided further evidences of the presence of α-galactose on the surface of *P. falciparum* sporozoites, based on α-galactose-binding *Bandeiraea* (*Griffonia*) *simplicifolia*-I isolectin IB_4_ labelling of sporozoite surfaces. Interestingly, they also demonstrated the protective effect against malaria associated to anti-α-galactose antibodies [19]. Thus, the UDP-galactose pool identified in the blood-stages of the parasite may possibly also be present in other life-stages and contribute to the biosynthesis of the proposed novel galactose-containing glycoconjugates [15]. This would also suggest the existence of at least one unidentified α-galactosyltransferase in the parasite genome.

## UDP-glucose

In eukaryotes UDP-glucose (UDP-Glc) is synthetized through an isomerization between glucose-6-phosphate (Glc6P) and glucose-1-phosphate (Glc1P) catalyzed by a phosphoglucomutase (PGM; EC 5.4.2.2). Glc1P is further activated to the sugar nucleotide generally by UTP-glucose-1-phosphate uridylyltransferase (UGP; EC 2.7.7.9) (Fig. 1). A homolog of PGM is present and expressed in the genome of *P. falciparum*, whereas the activation of Glc1P to UDP-Glc remains unknown as two enzymatic activities might be involved (see above): a UGP (EC 2.7.7.9), as in mammals [81] or a USP (EC 2.7.7.64), as in plants and *L. major* (Table 1) [15, 69, 82]. UDP-Glc levels in *P. falciparum* are relatively abundant in comparison to other sugar nucleotide pools identified on the blood stages [15]. The first evidences of UDP-Glc usage date back to 1994 when its incorporation/usage was detected in *P. falciparum* extracts [41].

A potential fate for UDP-Glc is the N-glycan-dependent quality control (QC) mechanism of glycoprotein folding. The mechanism consists of a UDP-Glc:glycoprotein glycosyltransferase (UGGT) and a Dol-P-Glc synthase, responsible for the biosynthesis of Dol-P-Glc precursors. UGGT normally glucosylates N-glycans of misfolded proteins in the ER in order to be recognized by the calreticulin/calnexin refolding system [83–87]. However, the N-glycan precursors synthetized by *P. falciparum* are constituted only by one or two GlcNAc residues missing the mannose residue that acts as UGGT acceptor. The parasite also lacks homologs for the components involved in this QC system [7, 88]. Another possible outcome for UDP-Glc may be the O-glucosylation of specific protein domains, such as epidermal growth factor (EGF) repeats [89] and/or the glucose substitution of O-fucose residues in TSR domains [90]. Interestingly, the crystal structures of HEK293T expressed recombinant CS and TRAP proteins shows both fucose and hexose residues attached to their TSR-domains [17, 18]. In any case, there are no clear candidates for glucosyltransferases in the parasite genome.

The UDP-Glc pool may also be related to the synthesis of glucose containing lipids. Glycolipids, as components of cellular membranes, play important roles in cell–cell contacts, membrane integrity and intracellular signaling [91–93]. In *P. falciparum*, glucose-containing lipids have been detected and an active glucosylceramide synthase activity (GCS; EC 2.4.1.80) has been identified in the parasite. This enzymatic activity adds glucose residues to dihydroceramide acceptors and is dependent on UDP-Glc [91, 94].

## Concluding remarks

There are still many challenges for the community to tackle when studying the glycobiology of *P. falciparum*. Glycosylation has always been a controversial issue in this parasite due to several reasons such as difficulties concerning the isolation and culturing of parasites; complications due to the interconnected nature of *P. falciparum* and its mammalian host cell membranes and structures; technical limitations of metabolic tracing through classical methodologies; and the lack of appropriate tools for genetic manipulation and culture methods standardization [9]. It seems clear that, besides GPI-anchors proteins, there is a limited scope for other types of glycosylation processes in *P. falciparum*, as compared to other protozoan parasites such as *Trypanosoma spp.* and *Leishmania spp.*, at least during the intracellular blood stages of the parasite. This may be due to the limited resources for *P. falciparum* within the relative biochemically inert red blood cell, including why there is a lack of sialyltransferase activities. Nonetheless, striking new pieces of evidence are emerging regarding overlooked glycosylation reactions that might be important for the parasite’s survival, infectivity and antigenicity. Furthermore, there is an obvious lack of knowledge about the presence and nature of parasite glycosylations during its extracellular stages.
